# Sociodemographic characteristics help predict Canadian urbanites’ acceptability of restaurant food environment policies

**DOI:** 10.3389/fnut.2024.1360360

**Published:** 2024-04-30

**Authors:** Jessica Lambert-De Francesch, Kadia Saint-Onge, Nazeem Muhajarine, Lise Gauvin

**Affiliations:** ^1^Centre de Recherche du Centre Hospitalier de l’Université de Montréal, Montreal, QC, Canada; ^2^Département de Médecine Sociale et Préventive, École de Santé Publique, Université de Montréal, Montreal, QC, Canada; ^3^Département de Kinésiologie, Faculté de Médecine, Université Laval, Québec City, QC, Canada; ^4^Department of Community Health and Epidemiology, College of Medicine, University of Saskatchewan, Saskatoon, SK, Canada; ^5^Saskatchewan Population Health and Evaluation Research Unit, Saskatoon, SK, Canada

**Keywords:** policy, acceptability, restaurant, food environment, sociodemographic factors, Canada

## Abstract

**Introduction:**

Public acceptability of policies aiming to improve the healthfulness of the restaurant food environment is key to their successful implementation. Yet, the acceptability of these policies remains ambiguous, especially across diverse population groups. This study aims to examine associations between sociodemographic characteristics and acceptability levels of three restaurant food environment policies of varying degrees of intrusiveness across 17 urban Canadian jurisdictions.

**Methods:**

Data was extracted from the THEPA survey, one of the largest and most jurisdictionally comprehensive surveys on intervention acceptability (*N* = 27,162). To account for potential jurisdictional differences in acceptability, for each policy, multilevel logistic regression models were developed.

**Results:**

Results indicated that, on average, those in complete agreement with the implementation of the targeted policies represented 20.3%–26.9% of participants, depending on the policy. Acceptability varied according to policy intrusiveness, jurisdiction, and participants’ sociodemographic characteristics. Women, individuals with household incomes of <$40,000/year, immigrants from a high-income country other than Canada, and Indigenous peoples were more likely to express complete agreement with all policies, versus men, participants with household incomes of $40,000–$79,999/year, Canadian-born individuals, and non-Indigenous individuals. A lower likelihood of expressing complete agreement with all policies was observed for those with a $80,000–$119,999/year household income, versus those with a $40,000–$79,999/year household income. For selected policies and models, other sociodemographic characteristics (i.e., age, education, and being born in a low-or middle-income country) predicted acceptability. The examined sociodemographic characteristics did not explain jurisdictional differences in acceptability.

**Discussion:**

Understanding jurisdictional differences in acceptability merits further research. Policy implications involve engaging diverse sociodemographic groups in conversations about acceptable ways in which their restaurant food environment could be rendered more healthful.

## 1 Introduction

Whether it be full-service restaurants or fast food outlets, restaurants, reputed for offering energy-dense and low nutritional quality foods (1, 2), represent a large proportion of food outlets in urban Canada (3). As the food environment (FE) both shapes and constrains modern day eating behavior (4), the current FE may be contributing to Canadians’ poor dietary habits and to the country’s heavy burden of diet-related chronic disease (5). Implementing FE policies, such as those targeting the restaurant industry, may be a promising strategy to facilitate healthy eating given that FE policies may alter the composition, labeling, marketing, retailing, pricing, and provision of foods (6). The implementation of FE policies may, however, be a challenging ordeal due to their low public acceptability (7). From an implementation science perspective, FE policy acceptability (i.e., agreement with the implementation of a FE policy) is of critical importance since policy acceptability will likely facilitate policy implementation and sustainability (8, 9). Moreover, citizens with high levels of acceptability for FE policies are more likely to advocate for healthier FE initiatives, which may act as a catalyst for policy implementation (10). Despite the importance of policy acceptability in the context of policy change, to date, the acceptability of FE policies, in general, and restaurant food environment (RFE) policies, in particular, remain poorly understood across diverse populations (11–15).

Person- and policy-related correlates of food policy acceptability have been the focus of two systematic reviews (12, 16). Regarding person-related correlates of food policy acceptability, both reviews report consistent trends across gender and age variables, with greater acceptability levels generally observed among women and older individuals. As for the associations between other sociodemographic variables (i.e., income, educational attainment, and race/ethnicity) and food policy acceptability, the evidence base is much more limited and contradictory. The dearth of studies including race and ethnicity variables is especially apparent, as neither of the systematic reviews provide information as to the size and direction of these associations. Only few individual studies have focused on Indigenous populations as well as culturally diverse populations, which have yielded heterogenous findings (8, 9, 11, 14, 17). Additionally, whether it be well established sociodemographic correlates of acceptability, like gender and age, or correlates that have been less extensively examined, like race and ethnicity, all sociodemographic-related associations are subject to methodological biases, with certain authors underscoring the utilization of convenience sampling, non-representative samples, and sometimes small sample sizes in the food policy acceptability literature (8, 13). Thus, even the links between well-established sociodemographic correlates of food policy acceptability would benefit from being replicated in more representative and larger samples.

In relation to policy-related correlates of food policy acceptability, both reviews highlight that food policy acceptability increases as a function of the policy’s stage of implementation and decreases as a function of the policy’s level of intrusiveness (12, 16). In other words, policies at more advanced stages of implementation and policies with lower levels of intrusiveness generally render high levels of acceptability. The latter relationship can notably be conceptualized using the Intervention Ladder (18), a model classifying policies from least to most intrusive: (1) opt for the status quo/ monitor the situation, (2) provide information, (3) enable choice, (4) change the default choice, (5) guide choice through incentives, (6) guide choice through disincentives, (7) restrict choice, and (8) eliminate choice.

Despite previously examining person- and policy-related correlates of acceptability for a wide range of policies, the current evidence base does not provide an in-depth exploration on how the above findings specifically relate to RFE policies. To the best of our knowledge, this specific angle has not been the primary focus of inquiry in any study thus far. Furthermore, the current body of literature delineating context specific variations, such as jurisdictional differences, in acceptability is scare, with only two identified Canadian studies including this dimension to their study focus (11, 19). These studies only provide limited data on acceptability of RFE policies.

In light of the incomplete evidence base and the methodological shortcomings identified in the literature, this study aims to estimate the direction and magnitude of relationships between sociodemographic characteristics (i.e., gender, age, education, household income, country of birth, and Indigenous status) and acceptability levels of three RFE policies of varying degrees of intrusiveness across 17 different urban Canadian jurisdictions. These three policies regard (1) changing the default side dish option in restaurant settings for a healthier alternative, (2) limiting the implementation of fast foods around schools, and (3) eliminating the offer of ultra-processed foods, such as chips, candy, and other unhealthy foods, in a wide array of municipal settings, namely restaurant settings.

## 2 Materials and methods

### 2.1 Participants and sampling

Participants were recruited as part of a large-scale survey titled targeting healthy eating and physical activity (THEPA) which examined the levels of acceptability for various built environment and policy interventions among 27,162 adults living in the 17 most densely populated census metropolitan areas (CMAs) in Canada. The THEPA survey is unique in its genre since it is among the largest and most jurisdictionally comprehensive datasets on intervention acceptability.

To take part in the THEPA study, participants had to be at least 18 years old, speak English and/or French, and agree to minimally share the first three digits of their six-digit residential postal code, confirming that their residence was indeed located in one of the 17 targeted CMAs. For 14 out of the 17 CMAs, target sample size was established at 1,200. However, to account for significantly higher population densities in Toronto, Montreal, and Vancouver, larger sample sizes were collected in these three CMAs. Participants could either respond to the survey electronically or verbally (by telephone). Regarding online surveys, participants were randomly selected from a pool of individuals who had previously completed questionnaires for both national and international survey firms. As for telephone surveys, participants’ telephone numbers were obtained via random digit dialing. Once contacted by telephone, participants were given the opportunity to respond either verbally or electronically. When participants preferred to fill out the questionnaire electronically, a survey link was sent to their personal email address. The questionnaires were administered in either English or French, the two official languages in Canada. All survey data was collected between October and December 2020, and all participants provided consent. The THEPA survey obtained ethics approval from the Ethics committee of the Centre Hospitalier de l’Université de Montréal on November 28th, 2019 (protocol number: 19.258).

### 2.2 Survey design and measures

The THEPA survey required participants to rate their level of agreement with the implementation of 45 built environment and policy interventions in their residential neighborhood (i.e., a 15-min walking distance from their residence). Among these interventions, the following three policies directly related to the RFE: (1) change the usual side dish in restaurants for a healthier option like salad instead of fries, (2) impose municipal regulations to limit fast food outlets around schools, (3) eliminate the offer of chips, candy, and other unhealthy foods in restaurants, cafeterias, and vending machines in municipal buildings like arenas and recreation centers. These policies directly relate to specific intervention categories established by the Intervention Ladder, respectively (1) change the default choice, (2) restrict choice, and (3) eliminate choice. Agreement levels were measured using a 4-point ordinal scale, ranging from completely agree to completely disagree, with a I don’t know/ I prefer not to answer option provided. A binary indicator was created to operationalize the acceptability level of each RFE policy (in this case, completely agree versus other). The rationale for examining those in complete agreement, in contrast to those responding any other case, was underpinned by the greater likelihood of those in complete agreement with the selected RFE policy to advocate for policy change.

Aside from investigating acceptability levels, the THEPA survey also inquired about participants’ lifestyle behaviors, neighborhood social cohesion level, and sociodemographic characteristics. The current study only focuses on the sociodemographic characteristics of participants. Gender was examined by asking participants if they identified as a man, woman, or other. These response options were then dichotomized into: man or woman. The responses for other were considered missing due to insufficient sample size. Age was measured by asking participants to indicate their year of birth. These responses were either recoded into: 18–34 years old, 35–54 years old, or 55 years old and over categories. Immigrant status was examined by asking participants to name their birth country. Responses were recoded into three categories: born in Canada, born in a high-income country (HIC) outside of Canada, or born in a low- or middle-income country (LMIC). This recoding was done according to the World Bank’s classification of countries according to their income level (20). Indigenous status was examined by asking participants if they identified as Indigenous or not. These response options were then dichotomized into: Indigenous or non-Indigenous. Educational attainment was examined by asking participants what their highest level of educational training was, where response options included: primary school, high school, trade school, college technical training, and university. These responses were recoded as one the following: high school or less, trade school or junior college, or university. Annual household income (gross) was examined by asking participants their annual household income before taxes, where response options were presented in $20,000 increments, ranging from < $20,000 to ≥ $160,000. Responses were assigned to one of the following categories: < $40,000, $40,000-$79,999, $80,000-$119,999, and ≥ $120,000. In this study, the reference group was comprised of non-Indigenous, Canadian-born women, aged 55 years and over, who had received university training, and whose annual gross household income was $40,000-$79,999. All sociodemographic questions included I don’t know / I prefer not to answer response option. These responses were considered missing.

### 2.3 Preliminary steps to analyses

To ensure population representativity, data was weighted using a two-fold procedure. First, to ensure that each CMA sample was reflective of the total CMA adult population, weights were applied to sex, age, and education variables. These weights were based on proportion estimates extracted from the 2016 Canadian census profile (21). Second, post-stratification weights were applied to all variables to account for CMA size differences. Curating the data also involved addressing missing data. To make full use of the dataset and to address biases which may result from data not missing at random, missing values were multiply imputed (22). Predictive mean matching methods enabled the prediction of missing values for all acceptability scores and sociodemographic characteristics. These predictions were made based on a pool of the 5 closest “donor” cases, where one of these cases was randomly selected to “donate” its value to the missing value case (23). The fact that predictive mean matching methods assign only observable cases to missing value cases constitutes their primary advantage over other imputation techniques (24). The process of assigning “donor” cases to missing data cases was carried out 10 times. Subsequently, one large, imputed dataset, pooling a total of 10 distinct imputed datasets, was created. All preliminary steps to analyses were conducted on version 28 of SPSS software.

### 2.4 Analyses: statistical modeling

Multilevel modeling (MLM) was applied to account for the hierarchical structure of the data. Contrary to linear multiple regression analyses, MLM does not violate the assumption of independence, as it recognizes that participants that were nested within the same CMA may have been more likely to respond alike due to shared contextual similarities (22, 25). To examine the association between acceptability levels of each policy and person level predictors (level 1), which were adjusted for CMA differences (level 2), multilevel logistic regression models were developed. Logistic models were chosen because the outcome variable was dichotomous (completely agree versus other) (26). The first step was to conduct binary multilevel logistic regression analyses to assess the relationship between each sociodemographic variable and the acceptability levels of each RFE policy. The second step involved creating null models, where CMA-related estimates of acceptability were included as random effects. The null model allowed for the estimation of the mean acceptability level of all CMAs (intercept) and the mean deviation of CMAs around the intercept (variance). The third step involved consecutively creating 4 distinct models, where level-1 predictors were included as fixed effects. The 4 models, respectively, included the following variables: (1) age and gender, (2) age, gender, and education, (3) age, gender, education, and income, and (4) age, gender, education, income, immigrant status, and Indigenous status. The order in which the variables were entered in the models was based on existing evidence, where variables with the most systematically observed associations were successively entered. All models were created using full maximum likelihood estimations. Additionally, for both null and full models (models 4), intercept values, plausible value ranges (i.e., magnitude of variation regarding completely agree responses across CMAs), variance values, and chi-square values were reported. For each bivariate and multivariate regression performed, standardized odds ratios (ORs) and 95% confidence intervals (95 % CIs) were reported. All the steps described in this paragraph were repeated for each outcome variable. All modeling steps were performed using HLM 8.0 software with weighted/imputed datasets as well as with weighted/non-imputed datasets.

## 3 Results

### 3.1 Participants

Table 1 presents the sociodemographic characteristics of participants using unweighted/non-imputed, weighted/non-imputed data, and weighted/imputed data. Both weighted datasets adjusted for the under/over representation of certain population groups. The imputed dataset corrected for the non-random missing value patterns, especially regarding income which was the variable with the highest proportion of missing values (11.3% of cases). In all, the weighted/imputed dataset provided the best representation of the Canadian urban population.

**TABLE 1:** Sociodemographic characteristics of the 27,162 survey respondents living in one of 17 targeted Canadian census metropolitan areas which provided data between October and December 2020.

Characteristic	Unweighted, non-imputed dataset	Weighted, non-imputed dataset	Weighted, imputed dataset
	***N* (%)**	**%**	**%**
**Gender**			
Male	11,962 (44.0)	47.9	48.2
Female	15,017 (55.3)	51.4	51.8
Other	72 (0.3)	0.3	–
Missing	111 (0.4)	0.4	–
**Age**			
18 to 34 years	6,992 (25.7)	28.9	29.5
35 to 54 years	8,881 (32.7)	34.4	35.1
55 years or older	10,669 (39.3)	34.3	35.4
Missing	620 (2.3)	2.4	–
**Education**			
High school or less	5,700 (21.0)	40.9	41.2
Trade school or junior college	8,779 (32.3)	29.3	29.6
University	12,372 (45.5)	28.6	29.2
Missing	311 (1.1)	1.2	–
**Annual family income before taxes**			
Less than $40,000	5,294 (19.5)	23.4	25.6
Between $40,000 and $79,999	8,020 (29.5)	29.5	31.9
Between $80,00 and $119,999	5,920 (21.8)	20.3	23.6
$120,000 and over	4,847 (17.8)	15.3	18.9
Missing	3,081 (11.3)	11.6	–
**Country of birth**			
Canada	20,917 (77)	75.1	77.9
High-income country outside of Canada	1,649 (6.1)	6.2	7.2
Low- or middle-income country	3,353 (12.3)	13.6	14.9
Missing	1,243 (4.6)	5.0	–
**Self-reported Indigenous status**			
Non-Indigenous	25,483 (93.8)	92.4	94.5
Indigenous	1,173 (4.3)	5.3	5.5
Missing	506 (1.9)	2.3	–

### 3.2 Policy acceptability levels

The proportion of missing data for acceptability levels accounted for 5.8% of responses for both the default choice and the eliminate choice policy. A total of 6.9% of acceptability responses were missing for the choice restriction policy. As per Table 2, across CMAs, only a minority of participants were in complete agreement with the proposed policies. More specifically, according to the weighted/imputed results for the null models (i.e., models with no level-1 predictor variables), the default choice policy obtained the highest proportion of completely agree responses (26.9%), while the eliminate choice policy obtained the lowest proportion (20.3%). The plausible value ranges for completely agree responses ranged from 23.2 to 30.5% for the default choice policy, from 21.6 to 30.8% for the restrict choice policy, and from 15.7 to 24.8% for the eliminate choice policy. As attested by chi-square test results (*p* < 0.001), inter-CMA differences in acceptability levels were deemed statistically significant for all policies, and this in both null and full models (i.e., models with all level-1 predictor variables). Intriguingly, when comparing variances before and after adjusting for sociodemographic characteristics, values did not decrease in the full models, comparatively to the null models (e.g., variance after sociodemographic adjustments for the default choice policy remained 0.01). This suggests that factors, other than those pertaining to sociodemographic characteristics, are to be accounted for when examining CMA- level differences in acceptability levels.

**TABLE 2:** Estimated proportions of complete agreement levels for three RFE policies, overall and across Canadian census metropolitan areas (CMAs), based on survey responses from 27,162 participants living in one of 17 targeted CMAs (October–December 2020).

Intervention	Change the usual side dish (i.e., default choice policy)	Limit fast food outlets around schools (i.e., restrict choice policy)	Eliminate the offer of chips, candy, and other unhealthy foods (i.e., eliminate choice policy)
**Null model results**			
Intercept (average acceptability level across CMAs)	Intercept: −1.01 Plausible value ranges: 23.2%–30.5% Overall estimate: 26.9% [Intercept: −0.98 Plausible value ranges: 25.6–32.49% Average: 29.0%]	Intercept: −1.05 Plausible value ranges: 21.6–30.8% Overall estimate: 26.2% [Intercept: −1.02 Plausible value ranges: 21.9–31.6% Average: 26.8%]	Intercept: −1.39 Plausible value ranges: 15.7–24.8% Overall estimate: 20.3% [Intercept: −1.36 Plausible value ranges: 16.3–25.4% Average: 20.8%]
Variance of CMAs around intercept (random effect)	Variance: 0.01 Significance level of chi-square: *p* < 0.001 [Variance: 0.01 Significance level of chi-square: < 0.001]	Variance: 0.02 Significance level of chi-square: *p* < 0.001 [Variance: 0.02 Significance level of chi-square: < 0.001]	Variance: 0.02 Significance level of chi-square: *p* < 0.001 [Variance: 0.02 Significance level of chi-square: < 0.001]
**Full model results (model 4)**			
Intercept (average acceptability level for individuals fitting the reference category criteria)	Intercept: −1.23 Plausible value ranges: 19.2–26.3% Overall estimate: 22.8% [Intercept: −1.23 Plausible value ranges: 19.5–26.0% Average: 22.7%]	Intercept: −0.97 Plausible value ranges: 23.0–32.6% Overall estimate: 27.8% [Intercept: −0.93 Plausible value ranges: 23.56–33.8% Average: 28.7%]	Intercept: −1.36 Plausible value ranges: 16.0–25.8% Overall estimate: 20.9% [Intercept: −1.33 Plausible value ranges: 16.3–26.2% Average: 21.3%]
Variance	Variance: 0.01 Significance level of chi-square: *p* < 0.001 [Variance: 0.01 Significance level of chi-square: *p* < 0.001]	Variance: 0.02 Significance level of chi-square: *p* < 0.001 [Variance: 0.02 Significance level of chi-square: *p* < 0.001]	Variance: 0.02 Significance level of chi-square: *p* < 0.001 [Variance: 0.02 Significance level of chi-square: *p* < 0.001]

Estimates presented without brackets represent pooled results from weighted/imputed datasets, whereas estimates in brackets represent results from weighted/non-imputed datasets.

### 3.3 Sociodemographic correlates of acceptability

Findings relating to sociodemographic differences in RFE policy acceptability levels appear in Table 3. Findings were relatively consistent across datasets (i.e., weighted/non-imputed and weighted/imputed). This section reports exclusively on results extracted from the weighted/imputed dataset, as these results are thought to most adequately depict reality.

**TABLE 3:** Results from (1) bivariate and (2) multivariate multilevel logistic regression analyses illustrating the relationships between sociodemographic variables and completely agree responses for three RFE policies, using both weighted/imputed and weighted/non-imputed data, based on responses from 27,162 survey respondents living in one of 17 Canadian census metropolitan areas (data collected between October–December 2020).

Intervention Variables	Change the usual side dish (i.e., default choice policy)					Limit fast food outlets around schools (i.e., restrict choice policy)					Eliminate the offer of chips, candy, and other unhealthy foods (i.e., eliminate choice policy)				
**Model** **ORweighted**/ **imputed** **95 % CI** **ORweighted**/ **non-imputed** **95 % CI**	**Biva-riate**	**Model 1:** **Gender** **+** **age**	**Model 2: Model 1** **+** **education**	**Model 3: Model 2** **+** **income**	**Model 4: Model 3** **+** **country of** **birth** **+** **Indigenous** **status**	**Biva-riate**	**Model 1: Gender** **+** **age**	**Model 2: Model 1** **+** **education**	**Model 3: Model 2** **+** **income**	**Model 4: Model 3** **+** **country of** **birth** **+** **Indigenous** **status**	**Biva-riate**	**Model 1: Gender** **+** **age**	**Model 2: Model 1** **+** **education**	**Model 3: Model 2** **+** **income**	**Model 4: Model 3** **+** **country of** **birth** **+** **Indigenous** **status**
**Gender**															
Man	1.00	1.00	1.00	1.00	1.00	1.00	1.00	1.00	1.00	1.00	1.00	1.00	1.00	1.00	1.00
Woman	**1.51** 1.43, 1.60	**1.51** 1.43, 1.60	**1.51** 1.43, 1.60	**1.50** 1.41, 1.58	**1.51** 1.43, 1.60	**1.17** 1.11, 1.24	**1.17** 1.10, 1.24	**1.18** 1.11, 1.25	**1.16** 1.10, 1.23	**1.17** 1.10, 1.24	**1.18** 1.11, 1.26	**1.18** 1.11, 1.26	**1.18** 1.11, 1.26	**1.17** 1.10, 1.25	**1.18** 1.11, 1.26
	**1.54** 1.42, 1.67	**1.54** 1.42, 1.67	**1.54** 1.42, 1.67	**1.52** 1.40, 1.65	**1.53** 1.42, 1.66	**1.19** 1.09, 1.30	**1.19** 1.08, 1.30	**1.19** 1.08, 1.31	**1.17** 1.07, 1.29	**1.18** 1.07, 1.29	**1.20** 1.13, 1.27	**1.19** 1.12, 1.27	**1.20** 1.13, 1.28	**1.19** 1.11, 1.28	**1.20** 1.12, 1.28
**Age**															
18 to 34 years	**0.92** 0.85, 0.98	0.93 0.86, 1.00	0.93 0.86, 1.00	0.93 0.86, 1.00	**0.90** 0.84, 0.97	**0.86** 0.80, 0.93	**0.87** 0.81, 0.93	**0.86** 0.80, 0.93	**0.86** 0.80, 0.93	**0.85** 0.78, 0.91	**0.87** 0.81, 0.95	**0.88** 0.81, 0.96	**0.88** 0.81, 0.95	**0.88** 0.81, 0.95	**0.84** 0.78, 0.92
	0.91 0.84, 1.00	0.92 0.85, 1.01	0.92 0.84, 1.01	0.92 0.85, 1.01	**0.90** 0.83, 0.98	0.85 0.67, 1.08	0.85 0.67, 1.09	0.85 0.66, 1.09	0.85 0.67, 1.08	0.83 0.65, 1.04	0.87 0.67, 1.14	0.88 0.67, 1.14	0.87 0.66, 1.15	0.87 0.66, 1.14	0.84 0.63, 1.10
35 to 54 years	0.99 0.92, 1.06	1.00 0.93, 1.07	0.99 0.92, 1.06	1.00 0.93, 1.08	0.99 0.92, 1.07	0.96 0.90, 1.03	0.97 0.90, 1.03	0.95 0.89, 1.01	0.96 0.90, 1.03	0.96 0.89, 1.02	**0.92** 0.85, 0.99	**0.92** 0.85, 0.99	**0.89** 0.83, 0.96	**0.90** 0.84, 0.98	**0.89** 0.82, 0.96
	0.99 0.92, 1.07	1.00 0.93, 1.08	0.99 0.92, 1.07	1.00 0.93, 1.08	1.00 0.92, 1.08	0.95 0.84, 1.06	0.95 0.84, 1.07	0.93 0.83, 1.04	0.94 0.85, 1.05	0.94 0.84, 1.05	**0.90** 0.82, 0.99	**0.90** 0.83, 0.99	**0.87** 0.80, 0.96	**0.88** 0.82, 0.96	**0.87** 0.80, 0.95
55 years or older	1.00	1.00	1.00	1.00	1.00	1.00	1.00	1.00	1.00	1.00	1.00	1.00	1.00	1.00	1.00
**Education**															
High school or less	0.94 0.88, 1.00		**0.93** 0.87, 0.99	**0.88** 0.82, 0.95	**0.89** 0.83, 0.96	**0.83** 0.78, 0.89		**0.83** 0.77, 0.89	**0.79** 0.73, 0.85	**0.78** 0.73, 0.84	**0.79** 0.73, 0.85		**0.78** 0.72, 0.84	**0.74** 0.68, 0.80	**0.75** 0.69, 0.82
	0.95 0.87, 1.04		0.94 0.87, 1.02	**0.89** 0.84, 0.96	**0.91** 0.84, 0.98	**0.83** 0.74, 0.93		**0.82** 0.73, 0.92	**0.77** 0.69, 0.86	**0.76** 0.68, 0.85	**0.79** 0.68, 0.92		**0.77** 0.67, 0.90	**0.74** 0.63, 0.87	**0.76** 0.65, 0.87
Trade school or junior college	1.03 0.96, 1.11		1.02 0.94, 1.09	0.99 0.92, 1.07	1.00 0.93, 1.08	**0.90** 0.83, 0.96		**0.89** 0.83, 0.96	**0.87** 0.81, 0.94	**0.87** 0.80, 0.93	0.93 0.86, 1.01		**0.92** 0.85, 0.99	**0.90** 0.83, 0.98	**0.91** 0.84, 0.99
	1.06 0.97, 1.17		1.04 0.95, 1.15	1.02 0.93, 1.12	1.03 0.96, 1.11	0.90 0.80, 1.00		**0.89** 0.79, 0.99	**0.87** 0.78, 0.97	**0.86** 0.78, 0.96	0.94 0.78, 1.14		0.93 0.77, 1.13	0.92 0.75, 1.14	0.93 0.77, 1.13
University	1.00		1.00	1.00	1.00	1.00		1.00	1.00	1.00	1.00		1.00	1.00	1.00
**Annual family income before taxes**															
Less than $40,000	**1.13** 1.05, 1.22			**1.14** 1.06, 1.24	**1.13** 1.04, 1.22	**1.11** 1.03, 1.20			**1.14** 1.06, 1.2	**1.13** 1.05, 1.22	**1.14** 1.05, 1.25			**1.19** 1.09, 1.30	**1.17** 1.07, 1.28
	1.13 0.88, 1.45			1.16 0.89, 1.50	1.14 0.88, 1.48	1.13 0.93, 1.39			1.18 0.96, 1.46	1.17 0.95, 1.44	1.14 0.96, 1.37			**1.20** 1.02, 1.42	**1.19** 1.01, 1.40
Between $40,000 and $79,999	1.00			1.00	1.00	1.00			1.00	1.00	1.00			1.00	1.00
Between $80,000 and $ 119,999	**0.90** 0.82, 0.98			**0.90** 0.83, 0.98	**0.90** 0.83, 0.99	**0.90** 0.82, 0.99			**0.89** 0.81, 0.97	**0.89** 0.81, 0.98	**0.90** 0.82, 0.99			**0.89** 0.81, 0.97	**0.89** 0.81, 0.98
	**0.88** 0.80, 0.98			0.90 0.81, 1.00	0.91 0.81, 1.01	0.90 0.80, 1.01			**0.89** 0.79, 0.99	**0.89** 0.79, 0.99	0.91 0.83, 1.00			0.91 0.82, 1.00	**0.91** 0.84, 0.99
$120,000 and over	0.94 0.86, 1.04			0.95 0.86, 1.04	0.96 0.87, 1.05	0.99 0.90, 1.09			0.95 0.87, 1.05	0.96 0.87, 1.06	1.03 0.93, 1.15			0.99 0.89, 1.11	1.01 0.90, 1.13
	0.94 0.82, 1.08			0.96 0.83, 1.12	0.98 0.85, 1.12	0.98 0.81, 1.19			0.94 0.78, 1.14	0.95 0.79, 1.14	1.07 0.88, 1.31			1.04 0.81, 1.33	1.05 0.84, 1.33
**Country of Birth**															
Canada	1.00				1.00	1.00				1.00	1.00				1.00
High-income country other than Canada	**1.34** 1.21, 1.49				**1.32** 1.19, 1.47	**1.22** 1.08, 1.37				**1.18** 1.05, 1.34	**1.39** 1.23, 1.57				**1.34** 1.18, 1.52
	**1.38** 1.24, 1.54				**1.37** 1.23, 1.53	**1.21** 1.08, 1.34				**1.16** 1.03, 1.31	**1.40** 1.19, 1.64				**1.34** 1.17, 1.54
Low- or middle-income country	**1.26** 1.15, 1.38				**1.25** 1.14, 1.37	1.09 1.00, 1.18				1.05 0.97, 1.15	**1.38** 1.25, 1.53				**1.34** 1.21, 1.48
	**1.24** 1.08, 1.44				**1.23** 1.03, 1.46	1.08 1.00, 1.17				1.04 0.93, 1.16	**1.40** 1.22, 1.60				**1.35** 1.15, 1.59
**Self-reported Indigenous status**															
No	1.00				1.00	1.00				1.00	1.00				1.00
Yes	**1.60** 1.42, 1.81				**1.68** 1.48, 1.90	**1.46** 1.29, 1.65				**1.55** 1.37, 1.76	**1.57** 1.39, 1.77				**1.66** 1.47, 1.88
	**1.58** 1.21, 2.07				**1.66** 1.29, 2.13	**1.49** 1.18, 1.89				**1.60** 1.31, 1.95	**1.60** 1.34, 1.91				**1.70** 1.50, 1.93

Estimates presented at the top of each cell represent pooled results from weighted/imputed datasets, whereas estimates presented at the bottom of each cell represent results from weighted/non-imputed datasets. Bold values indicate the statistically significant findings.

#### 3.3.1 Change the usual side dish (i.e., default choice policy)

Differences in acceptability levels for the default choice policy were found according to all sociodemographic characteristics. Women were 1.51 times [95% CI: 1.43, 1.60] more likely to be in complete agreement with this policy than men, and this in both the bivariate and full model. In both bivariate and full models, participants aged 18–34 years old were less likely to express complete agreement for the targeted policy than those aged 55 years old and more, e.g., OR_*bivariate*_: 0.92 [95% CI: 0.85, 0.98]. As for education variables, in the bivariate model, no statistically significant association was found between high school training (or less) and acceptability levels for the default choice policy. Yet, in models 2, 3, and 4, those with a high school training or less had a statistically significant lower likelihood of being in complete agreement with the proposed policy than those with university training (e.g., OR_*model4*_: 0.89 [95% CI: 0.83, 0.96]). With the exception of the $120,000 income category, statistically significant associations were noted for all income brackets, and this irrespective of the model. Participants whose household earned less than $40,000 per year were more inclined to be in complete agreement with the policy than those whose household earned $40,000-$79,999 per year (e.g., OR_*bivariate*_: 1.13 [95% CI: 1.05, 1.22]). Conversely, participants falling within the $80,000-$119,999 income bracket consistently had lower odds of being in complete agreement with this policy than those belonging to the $40,000-$79,999 reference category (e.g., OR_*bivariate*_: 0.90 [95% CI: 0.82, 0.98]). In all models, both those born in a HIC other than Canada and those born in a LMIC had greater odds of expressing complete agreement for the targeted policy than their Canadian-born counterparts (e.g., OR_*bivariate*_: 1.34 [95% CI: 1.21, 1.49]; OR_*bivariate*_: 1.26 [95% CI: 1.15, 1.38], respectively). Comparatively to non-Indigenous individuals, Indigenous participants were more likely to be in complete agreement with this policy, regardless of the model (e.g., OR_*bivariate*_: 1.60 [95% CI: 1.42, 1.81]).

#### 3.3.2 Limit fast food outlets around schools (i.e., choice restriction policy)

Sociodemographic predictors of acceptability levels for the limiting fast food outlets around schools policy closely resembled those of the default choice policy. Similarly to previously stated results, across all models, women were more likely to be in complete agreement with the targeted policy than men (e.g., OR_*bivariate*_: 1.17 [95% CI: 1.11, 1.24]). Those aged 18–34 years old were also less likely to be in complete agreement with this zoning policy, contrary to their counterparts aged 55 years and over, and this in both bivariate and all multivariate models (e.g., OR_*bivariate*_: 0.86 [95% CI: 0.80, 0.93]). Much like the results from the first policy, it was less probable for those with a high school level training (or less) to exhibit complete agreement levels with the targeted policy than those with university training, and this regardless of the model (e.g., OR_*bivariate*_: 0.83 [95% CI: 0.78, 0.89]). However, unlike the first policy, those with a trade school or junior college training were also less likely to express complete agreement comparatively to the reference category, and this across all models (e.g., OR_*bivariate*_: 0.90 [95% CI: 0.83, 0.96]). As for income, comparable results with the first policy were once more obtained, where those within the lowest income bracket were consistently more likely to express complete agreement with the selected policy than those within the 40,000$–79,999$ income bracket (e.g., OR_*bivariate:*_ 1.11 [95% CI: 1.03, 1.20]). An association, in the opposite direction, was observed in all models for those whose family income was between $80,000-$119,999 (OR_*bivariate*_: 0.90 [95% CI: 0.82, 0.99]). As for country of birth, results generally mirror previous findings, where those born in a HIC other than Canada had greater odds of displaying complete levels of agreement for the restrict choice policy, and this across all models (e.g., OR_*bivariate*_: 1.22 [95% CI: 1.08, 1.37]). As for those born in a LMIC, this time, no statistically significant association was found in both bivariate and multivariate models. Finally, results for Indigenous status converge with the first policy, where those with an Indigenous status were, regardless of the model, more likely to be in complete agreement with the zoning policy than their non-Indigenous homologs (e.g., OR_*bivariate*_: 1.46 [1.29, 1.65]).

#### 3.3.3 Eliminate the offer of chips, candy, and other unhealthy foods (i.e., eliminate choice policy)

The direction and magnitude of associations between sociodemographic characteristics and acceptability levels for the eliminate choice policy nearly matched all results from the previous two policies. Analogous to previous results, irrespective of the model, women, those whose household earnings were less than $40,000 per annum, those from a HIC other than Canada, and those with an Indigenous status were all more likely to be in complete agreement with the eliminate choice policy, comparatively to men, those belonging to the $40,000-$79,999 annual income category, those born in Canada, and those who identify as non-Indigenous. For instance, the ORs in the bivariate models for these variables were, respectively: 1.18 [95% CI: 1.11, 1.26] for women, 1.14 [95% CI: 1.05, 1.25] for those earning less than $40,000 per annum, 1.39 [95% CI: 1.23, 1.57] for those from a HIC other than Canada, and 1.57 [95% CI: 1.39, 1.77] for Indigenous status individuals. Also mirroring the two former policies, those whose household annual income was between $80,000-$119,999 had lower odds of completely agreeing with the eliminate ultra-processed food policy than those whose household annual income was $40,000-$79,999, and this independently of the model examined (e.g., OR_*bivariate*_: 0.90 [95% CI: 0.82, 0.99]). As for age variables, the direction and magnitude of associations between acceptability levels and being aged 18–34 years old were nearly identical to those in the restrict choice policy. Indeed, across all models, participants in the 18–34 years old age category were less likely to be in complete agreement with the eliminate choice policy than those belonging to the 55 years and older category (e.g., OR_*bivariate*_: 0.87 [95% CI: 0.81, 0.95]). Unique to this targeted policy, those aged between 35 and 54 years old were also found to have lower odds of being in complete agreement with the choice elimination policy than those belonging to the 55 years and older category, and this in all models (e.g., OR_*bivariate*_: 0.92 [95% CI: 0.85, 0.99]). Furthermore, unlike the default choice policy, but akin to the restrict choice policy, high school level training or less predicted acceptability levels in all models, with the bivariate OR being 0.79 [95% CI: 0.73, 0.85], in contrast to those with university education. Similarly to the restrict choice policy, trade school or junior college training also predicted acceptability levels. However, this association was only observed in models 2, 3, and 4 (e.g., OR_*model4*_: 0.91 [95% CI: 0.84, 0.99]). Lastly, in all models, those from a LMIC were more likely to be in complete agreement with the eliminate choice policy than Canadian-born participants, akin to the acceptability levels for the default choice policy (e.g., OR_*bivariate*_: 1.38 [95% CI: 1.25, 1.53]).

## 4 Discussion

In this study, we estimated the direction and magnitude of relationships between sociodemographic characteristics and acceptability levels for three RFE policies of varying degrees of intrusiveness across 17 different urban Canadian jurisdictions.

Our results suggest that only a minority of respondents expressed complete agreement with the targeted policies. The policy that rendered the highest proportion of completely agree responses was the default choice policy, whereas the eliminate ultra-processed food policy had the lowest overall proportion of these responses. Statistically significant differences in acceptability levels were observed across CMAs. These differences persisted even after adjusting for sociodemographic variables. When examining sociodemographic predictors of acceptability, consistent patterns were observed across policies and models. Our results suggest that women, those with a gross household income less than $40,000 per annum, those born in a HIC outside of Canada, and those with an Indigenous status had higher odds of being in complete agreement with all examined policies than those fitting the reference category criteria. Across all policies and models, those within the $80,000-$120,000 annual income category were less likely to be in complete agreement with the targeted policies than those within the income reference category. For selected policies and models, those (1) aged 18–34 years old, (2) aged 35–54 years old, (3) with a high school training (or less), and (4) with a trade school or junior college level training were less inclined to be in complete agreement with the targeted policies, comparatively to those aged 55 years and over and those with university training. For two out of the three policies, being born in a LMIC was associated with higher odds of being in complete agreement with the targeted policies, comparatively to participants born in Canada.

Our results regarding variations in acceptability levels, based on examined policy, align with what is proposed by the Intervention Ladder, where an inverse relationship between policy acceptability and policy intrusiveness is observed (12, 16). Unique to our study, we were also able to show jurisdictional differences in acceptability levels. To this effect, our results oppose those of Kongats et al. (19) who found no statistically significant differences in support levels for selected RFE policies (e.g., obligatory restaurant menu labeling) among policy influencers residing in either Alberta or Quebec, two Canadian provinces. Our results also do not support those of Bhawra et al. (11) who found no statistically significant differences in support levels for RFE policies, like fast food zoning restrictions, among residents aged 16–30 years old from five Canadian cities. The latter authors’ results may; however, not be comparable to ours due to differences in targeted population. In addition to observing differences across CMAs, we were also able to show that jurisdictional differences in acceptability levels persisted even after controlling for sociodemographic variables. This suggests that additional factors, such as other person- or context-related factors, may be responsible for explaining CMA-level differences in acceptability levels. Future research is warranted to explain these differences.

As for sociodemographic differences in acceptability levels, our results are especially coherent with previous gender-based findings (12, 16). Women’s greater odds of being in complete agreement with the targeted policies may be attributable to their overall greater levels of health consciousness (27) and their greater desire to adhere to Western society’s beauty ideals based on thin or fit body phenotypes (28). The links between the latter attributes and women’s greater likelihood of being in complete agreement with the targeted policies are, however, only speculative. Further research is needed to explain these associations. Regarding education variables, despite our results not being fully consistent, our findings generally tie in with previous findings showing that those with greater educational attainment may be more likely to display higher acceptability levels for food-related policies (12). This link may be explained by the generally greater health literacy levels observed among more educated individuals (29), health literacy previously being pinpointed as a positive predictor of acceptability (11). Yet, more research is needed to support the potential mediating effect of health literacy between educational attainment level and RFE acceptability levels. Greater health literacy and greater interest in health may also potentially help explain why individuals aged 55 years and older were generally more likely to be in complete agreement with the three targeted policies, comparatively to certain other younger groups (12, 16). As for our income-based findings, our results for those making less than $40,000 per year are somewhat unsurprising given the rising cost of healthy foods (30). As lower income households are more vulnerable to food insecurity (31), notably limiting their access to nutrient-dense foods, these individuals may want more food policies prioritizing the accessibility and availability of healthy food options. For individuals of higher income households, these policies may not be as crucial, as they may have fewer financial barriers to eating healthfully. The findings regarding immigrant status are challenging to interpret given the heterogeneity of participants’ birth country. We posit that these findings may be interpreted using the previously discussed linkage between food policy acceptability and food policy implementation stage. In this sense, prior residency in foreign cities where RFE policies have already been implemented may translate into greater acceptability levels for these now Canadian urbanites. However, more research is needed to fully understand immigrant-related differences in acceptability levels. Regarding the associations between Indigenous status and acceptability levels, the dearth of studies including this sociodemographic variable offers very little insight to interpret the observed attitudinal differences (11). More research would also be needed to substantiate these links.

This study makes an important scientific contribution to the food policy acceptability literature. This study’s novelty stems from its unique inclusion of selected RFE policy items, and it may be the first nationwide study to examine the acceptability of RFE policies across various sociodemographic groups. Methodological strengths include the utilization of a large and representative sample, the recognition of a multilevel data structure, and the utilization of imputation techniques aimed at reducing biases engendered by missing data. Despite this study’s innovative character and robust methods, its limitations are to be mentioned. The lack of specificity in the wording of the selected RFE policies may have led to an over/under reporting of acceptability levels. For example, some participants may be in complete agreement with changing the default side dish in fast food restaurants but hold a less favorable opinion for other restaurant settings, such as fine dining restaurants. Thus, although our results present a global portrait of acceptability levels of RFE policies, future research ought to examine if and how acceptability levels vary according to restaurant type. Another limitation warranting attention is the fact that this study did not account for the presence/absence of the proposed RFE policies within each participant’s area of residence. Future research ought to control for policy stage of implementation, as this has been found to help modulate public opinion (12, 16).

### 4.1 Policy implications

The disparities in RFE acceptability levels according to jurisdictional and sociodemographic characteristics highlight the need for more extensive and inclusive conversations about acceptable ways in which the urban RFE may be rendered more healthful. Creating further opportunities for dialog between community members, policymakers, and public health practitioners may enable a better understanding of the underlying drivers of RFE acceptability levels. By diligently considering the needs, desires, and concerns of all community members during the policymaking process, stakeholders would be in a better position to propose socially, culturally, and economically informed RFE policies. To this effect, community-informed decision making, akin to what is proposed here, not only enhances the relevance of proposed policies, but also leads to better community leadership, capacity, and vitality (32).

## 5 Conclusion

In conclusion, only a small proportion of participants expressed complete agreement levels for the three RFE policies, with more intrusive policies rendering lower acceptability levels than less intrusive ones. Acceptability levels significantly varied according to CMA and persisted even after controlling for sociodemographic variables. Our analyses based on sociodemographic characteristics indicated that certain population groups were more likely to express complete agreement with selected RFE policies. More research is needed to elucidate other individual and contextual factors predicting the acceptability levels of RFE policies. Additionally, further ongoing discussions with the public are needed to ensure that implemented policies best reflect the needs and desires of all community members.

## Data availability statement

The data analyzed in this study is subject to the following licenses/restrictions: the dataset analyzed in this article is not readily available because it contains potentially identifiable data. Requests for collaborative projects should be directed to LG and NM. Requests to access these datasets should be directed to lise.gauvin.2@umontreal.ca; nazeem.muhajarine@usask.ca.

## Ethics statement

The studies involving humans were approved by the Ethics committee of the Centre Hospitalier de l’Université de Montréal (protocol number: 19.258, approved on November 28th, 2019). The studies were conducted in accordance with the local legislation and institutional requirements. The participants provided their written informed consent to participate in this study.

## Author contributions

JLDF: Conceptualization, Data curation, Formal analysis, Methodology, Visualization, Writing – original draft, Writing – review & editing. KS-O: Conceptualization, Investigation, Writing – review & editing. NM: Conceptualization, Funding acquisition, Investigation, Project administration, Supervision, Writing – review & editing. LG: Conceptualization, Data curation, Funding acquisition, Investigation, Methodology, Project administration, Supervision, Validation, Writing – review & editing.
